# Oxidative Stress in Disease and Aging: Mechanisms and Therapies 2018

**DOI:** 10.1155/2018/2835189

**Published:** 2018-09-23

**Authors:** Claudio Cabello-Verrugio, Cristian Vilos, Raquel Rodrigues-Diez, Lisbell Estrada

**Affiliations:** ^1^Universidad Andres Bello, Santiago, Chile; ^2^Universidad Autónoma de Madrid, Spain; ^3^Universidad Bernardo O'Higgins, Santiago, Chile

Among the key factors influencing human health are the chronic diseases and aging, which have been increasing in the last decades. These pathological states are produced by several and diverse causes, and a common factor involved in most of them is oxidative stress. Cellular oxidative stress is defined as an imbalance between oxidative status, mainly by the formation of reactive oxygen species (ROS), and antioxidant defense mechanisms. Due to the broad and profound biological effects of ROS, in the last years, numerous experimental and clinical studies have focused their attention on the participation of oxidative stress as a key regulator in chronic pathological status and aging. Regarding this relationship, the aim of many ongoing studies is to elucidate the underlying mechanisms and role of oxidative stress in disease onset and development. In particular, there is an emphasis on finding new therapeutic strategies for chronic diseases and aging by decreasing oxidative stress.

This version of the annual special issue of “Oxidative Stress in Disease and Aging: Mechanisms and Therapies 2018” presents novel and relevant research regarding the mechanisms by which oxidative stress induces damage in the contexts of diseases and aging. Focus is given to the use of novel antioxidant strategies. The manuscripts within this special issue are all equally recommended by the editors, but the following contain especially interesting points worth comment.

G. Rowicka et al. evaluated the intensity of oxidative processes and the efficiency of antioxidant defense in children with celiac disease. The results do not shown differences; however, they demonstrated that the strict adherence to a gluten-free diet by children with celiac disease seems to be important for maintaining oxidative-antioxidant balance.

J. Ábrigo et al. shown a review of the mechanisms involved in the development of cachexia and their close relation and fine regulation of oxidative stress. The wide vision of the process and how oxidative stress is a key factor that can be targeted to treat the muscular dysfunction secondary to chronic diseases and aging are clearly presented in this review.

W. B. Jang et al. developed a novel antioxidant molecule, a novel antioxidant defined as MHY-1684, and studied its effect on the therapeutical effect of resident cardiac progenitor cells (CPCs) in diabetic cardiomyopathy. The authors demonstrated that the treatment with MHY-1684 may affect the activation and inhibition of mitochondrial dynamics-related signaling and mitochondrial function in response to ROS stress. In addition, the data allow concluding that the novel compound MHY-1684 acts as an ROS scavenger and might provide an effective therapeutic strategy for CPC-based cell therapy against diabetic cardiomyopathy.

C. He et al. investigated the effect of the antioxidant edaravone on the cardiac dysfunction during sepsis. The study showed that in a rat model of septic myocardial dysfunction, edaravone suppressed oxidative stress and protected the heart against septic myocardial injury and dysfunction through the induction of the HIF-1*α*/HO-1 pathway.

All of these highlighted studies, as well as the other manuscripts contained in this special issue, advance improvements on pathological statuses by using diverse antioxidant strategies. We firmly believe that these findings will be of relevance to research concerning OS, chronic diseases, and aging.

